# Supplementary material for: The increase in cell volume and nuclear number of the koji-fungus Aspergillus oryzae contributes to its high enzyme productivity
Source: eLife. 2025 Sep 23;14:RP107043. doi: 10.7554/eLife.107043 (PMC12456952; doi:10.7554/eLife.107043)
Supplement: Supplementary file 7. [file elife-107043-supp7.docx]

Supplementary file 7. Composition of Minimal medium

Minimal Medium

Glucose 10 g

NaNO3 6 g

KH2PO4 1.52 g

KCl 0.52 g

MgSO4・7H2O 0.52 g

Hunter's Trace element 2 mL

pH 6.5

per litter

Hunter's Trace element

ZnSO4・7H2O 2.2 g

H3BO3 1.1 g

MnCl2・4H2O 0.5 g

FeSO4・7H2O 0.5 g

CoCl2・6H2O 0.16 g

CuSO4・5H2O 0.16 g

(NH4)6Mo7O24・4H2O 0.11 g

per 100 mL
